# Multi-center study for acupuncture combined with Chinese medicine in the treatment of chronic spontaneous urticaria based on the theory of taking shu-stream points when the disease is aggravated

**DOI:** 10.1097/MD.0000000000021266

**Published:** 2020-08-14

**Authors:** Yuesi Qin, Jing Guo, Pan Song, Tianshu Hou, Yan He, Ming Han, Qianying Yu, Wenxia Lin, Mingling Chen, Hong Su

**Affiliations:** a Hospital of Chengdu University of Traditional Chinese Medicine; b Chengdu Integrated TCM & Western Medicine Hospital; c The First People's Hospital of Longquanyi District Chengdu; d Xinjin County Hospital of traditional Chinese Medicine, Chengdu, Sichuan, China.

**Keywords:** acupuncture, chronic spontaneous urticaria, clinical trial, Danggui Yinzi, shu-stream acupoints

## Abstract

Chronic spontaneous urticaria (CSU) is a common dermatologic disease that seriously affects patient quality of life. The choice of therapy to control the disease and prevent its recurrence has always presented a difficult clinical issue. Previous studies have shown that traditional Chinese medicine is a safe and effective treatment for CSU. Recently, the temporal rhythms of CSU, a disease characterized by intermittent flares of active disease and periods of little or no disease, have attracted the attention of traditional Chinese medicine researchers. We designed a multicenter, randomized, controlled study to evaluate the efficacy and safety of combining a Chinese herbal formulation with acupuncture using shu-stream acupoints applied on the corresponding time meridians during disease exacerbations. We plan to recruit 111 outpatients with CSU aged 18 to 65 years. Participants will be randomized to 1 of the 3 groups: group A, which will be given basic acupuncture and the herbal formulation dangui yinzi; group B, which will be given danggui yinzi and shu-stream acupuncture; and a control group, which will be given danggui yinzi alone. Patients will be treated for 4 weeks and followed for 8 additional weeks. Investigators will evaluate the following parameters: the symptoms and side effects of treatment, quality of life (using the chronic urticaria quality of life questionnaire), and overall patient condition. Each week, patients will also complete the measurement of 7-day urticarial activity score. This is the first use of a combination of shu-stream acupoints and Chinese herbal medicine in the treatment of CSU. If successful, it will prove to be a simple, inexpensive, treatment strategy for solving a difficult clinical problem.

## 1 Introduction

Chronic spontaneous urticaria (CSU) is an intractable skin disease characterized by intense itching, wheals, and/or angioedema. The disease is defined as having continuous or intermittent symptoms for more than 6 weeks,^[1,2]^ with no specific, identifiable trigger. CSU can seriously affect the patient's physical and mental health and has been associated with anxiety, depression, insomnia, and metabolic syndrome.^[3]^ Its impact on the quality of life is comparable to the impact of having severe coronary artery disease and even exceeds that associated with respiratory allergies.^[4]^

The prevalence of CSU has been reported to be about 0.5% to 1%,^[5–7]^ with a peak incidence between the ages of 20 and 40 years.^[8]^ Women are affected twice as often as men.^[9]^ The etiology of CSU is difficult to determine, but it has been reported to be related to food, medicine, physical factors, vitamin D deficiency,^[10]^ stress,^[2,11]^ chronic occult infection,^[12]^ and autoimmune diseases. The pathological mechanisms responsible for CSU have not been fully elucidated, but mast cells are key effector cells in CSU pathogenesis, and the activation and degranulation of these cells are still the central link in the pathogenesis of urticaria. Mast cells can be activated by immune and nonimmune mechanisms.^[13]^ At present, it is thought that CSU is rarely mediated by allergen-induced, type I allergic reactions, and that a histamine-independent inflammatory reaction is the basis of antihistamine treatment resistance.^[14,15]^ Indeed, the routine use of second-generation H1 antihistamines (sgAH) is recommended as the first-line treatment for CSU.^[16]^ However, a number of studies have shown that even if the dose is increased by 4-fold, the efficacy is not satisfactory.^[17]^ Omalizumab has been shown to be effective in treating some refractory urticaria, but its expensive cost cannot be ignored, and there may be long-term adverse effects.^[18,19]^ Currently, there is no effective treatment for all patients; therefore, it is necessary to explore and evaluate new therapies to provide more treatment options for CSU.

Traditional Chinese medicine (TCM) has unique advantages in the treatment of CSU. The classic herbal formulation danggui yinzi has been widely used clinically to treat the TCM diagnosis of blood deficiency and wind dryness, and it has shown definite efficacy and a good safety profile in this application.^[20,21]^ In addition to Chinese herbal medicine, acupuncture is an important part of complementary and alternative medicine, and studies have demonstrated its effectiveness and safety in treating CSU.^[14,22,23]^

Clinically, CSU is characterized by intermittent episodes of active disease (flares) and periods of little or no disease (remission), and both clinical and experimental studies have found that the level of disease activity is affected by temporal rhythms (ie, disease activity is exacerbated at predictable times during a 24-hour period).^[24,25]^ So, we posit a bold hypothesis: whether CSU can be more effectively treated if these temporal rhythms are considered when determining treatment options. Chinese medical literature supports this theory, stating, “If the disease alleviated sometimes and aggravated sometimes, select Shu-Stream Points.”^[26]^ Therefore, our multicenter study will combine an oral Chinese herbal formulation with the application of time-specific, shu-stream acupoints to determine if this approach can effectively relieve the clinical symptoms of urticaria. This will not only serve to expand the compendium of available external TCM treatments, but it will also provide new areas for research into the treatment of urticaria.

## 2 Methods

### 2.1 Study design

**Figure 1:**
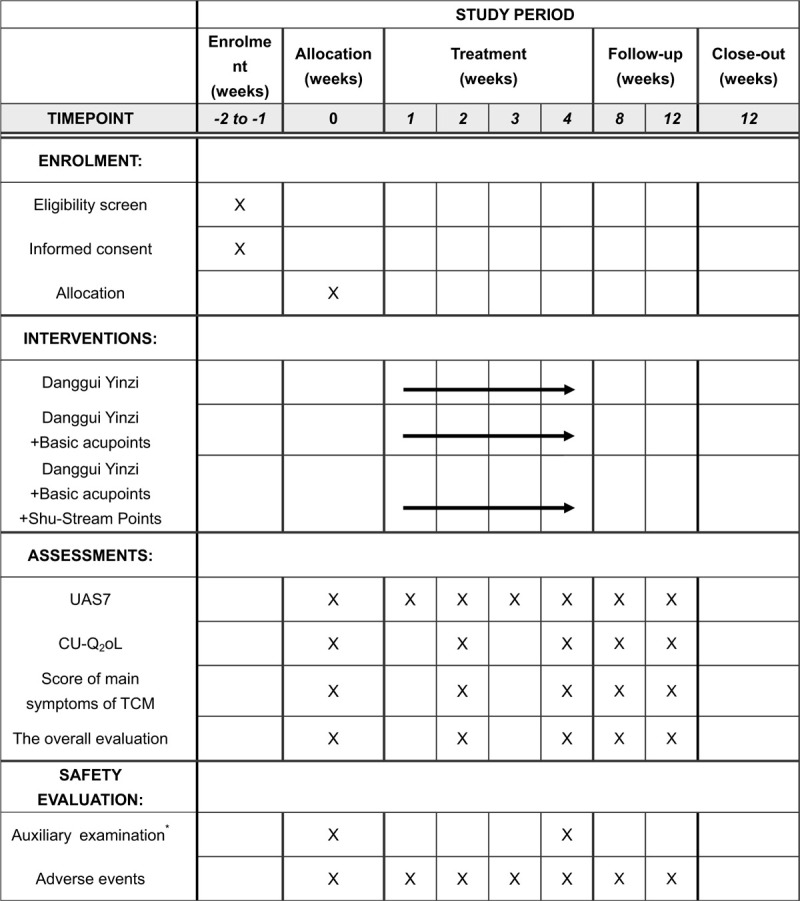
Spirit figure of enrollment, interventions, and assessments. UAS7 = 7-day urticaria activity score, CU-Q2oL = Chronic urticaria quality of life question-naire. ^∗^Auxiliary examination: blood test, kidney and liver function tests, and electrocardiogram.

**Figure 2:**
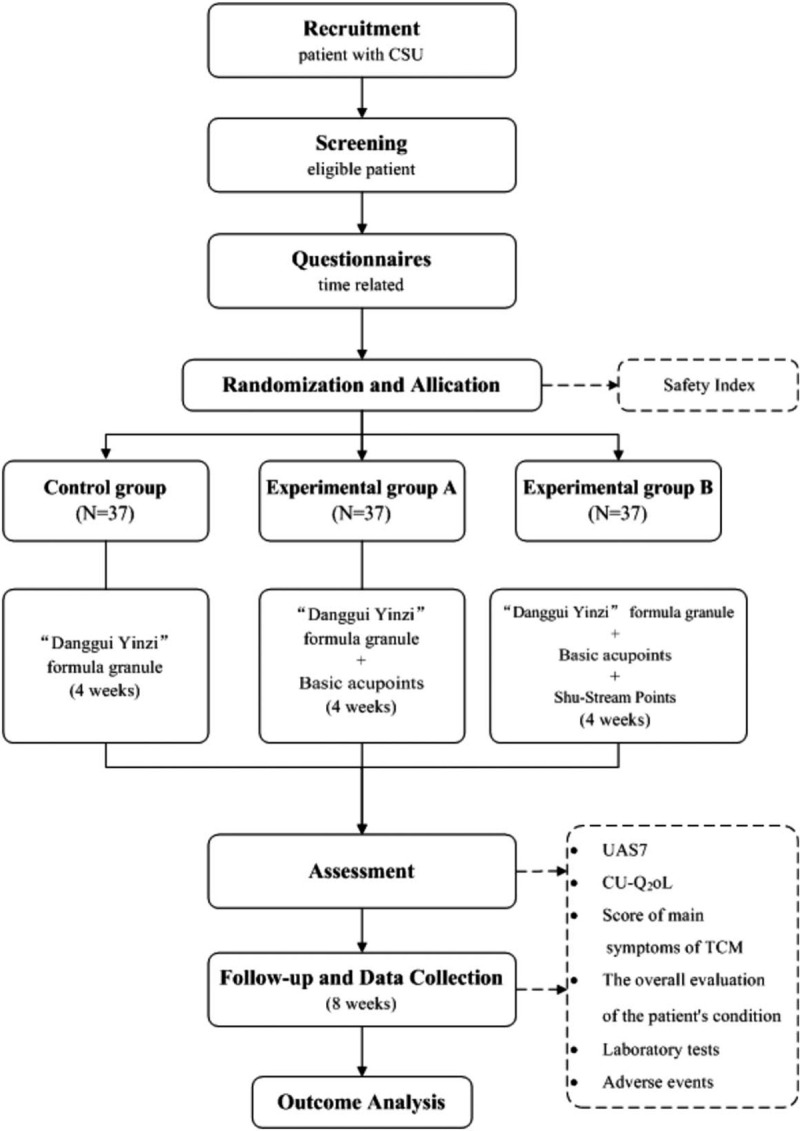
Flow chart of the study design.

This study is a multicenter, parallel-group, randomized controlled trial (RCT). The protocol was registered on May 3, 2020, in the Chinese Clinical Trial Registry as Clinical Trial No. ChiCTR2000032609. A total of 111 eligible participants will be enrolled and randomly assigned to the control group or 1 of the 2 treatment arms in a ratio of 1:1:1. Figure 1 shows the study registration schedule, interventions, and assessments. Figure 2 is a flow chart of the study.

### 2.2 Ethics approval

The study is in compliance with the Declaration of Helsinki (Edinburgh 2000 version). The study protocol has been approved by the (No. 2020KT-052). The personal data of the subjects will be kept confidential during the whole trial. Participants are completely voluntary to participate in the clinical research. At any stage of the research, they have the right to withdraw unconditionally, and their medical treatment and rights shall not be affected.

### 2.3 Recruitment

The participants will be recruited from the public through Chengdu Integrated TCM & Western Medicine Hospital and 3 subcenters, including the Affiliated Hospital of Chengdu University of TCM, The First People's Hospital of Longquanyi District Chengdu, and Xinjin Hospital of TCM. Before signing informed consent forms, participants will be screened first, and be aware of all the details of the clinical trial and the potential risks.

### 2.4 Sample size calculation

The following formula was used to estimate the sample size with the ability to perform multiple sample rate comparisons^[27]^: 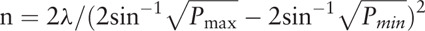


According to the estimation of effective rate,^[28–30]^ the maximum and minimum estimated overall rate of TCM in the treatment of CSU are 95% and 60%, respectively. According to the above formula, the sample size for each group is 33. Thus, assuming that 15% of patients are likely to be lost during follow-up, a total number of study samples is determined to be 111 cases.

### 2.5 Randomization

The central randomization method will be adopted, and Chengdu Integrated TCM & Western hospital will be responsible for the central randomization and data management. The random code of each center is obtained according to the order of “random number assigned by central code” generated by SAS statistical software. The results of the random sequence will be hidden in opaque envelopes.

### 2.6 Blinding

Because the interventions are different in each of the 3 groups, it is impossible to blind this study. Independent third parties will evaluate the patients and the study results.

### 2.7 Eligibility criteria

#### 2.7.1 Inclusion criteria

Participants who meet the following criteria will be included in the study: Adults aged 18 to 65 years who meet the diagnostic criteria for episodic CSU as specified in 2 different systems—the 2018 Chinese guidelines for urticaria and the 2017 European guidelines for urticaria (Table 1).^[1,14]^ They must also meet the TCM diagnostic criteria for blood deficiency and wind dryness syndrome (Table 2).^[31,32]^ Two designated attending TCM physicians will independently assess the participants to make this diagnosis.

**Table 1:**
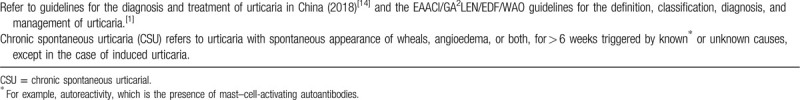
Western medicine diagnostic criteria for CSU.

**Table 2:**
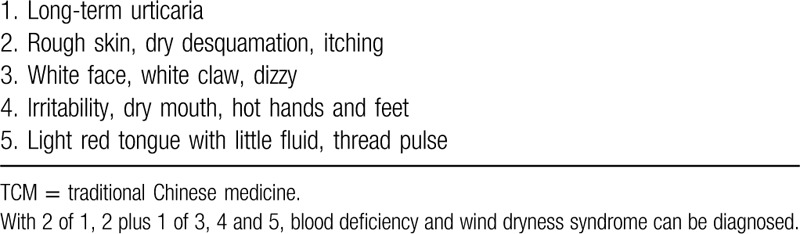
Diagnostic criteria for the TCM differentiation of blood deficiency and wind dryness syndrome.^[31,32]^.

Participants cannot have used any glucocorticoids, immunosuppressants, antihistamines, or other drugs within 1 month of beginning the study; and they cannot have had acupuncture for urticaria during the same time period. They must be free of any psychological or mental illness, have no communication barriers, and be able to sign the informed consent voluntarily and accept and comply with the treatment as specified in the protocol.

#### 2.7.2 Exclusion criteria

Patients who meet the following criteria will be excluded from participation in the study: People with severe cerebrovascular disease or disease of the liver, kidneys, or other organs, or those with systemic diseases and malignant tumors; pregnant or lactating women, or women of childbearing age who plan to become pregnant during the study period; patients with comorbid infections, severe gastrointestinal symptoms, laryngeal edema, or other potentially life-threatening systemic symptoms. Patients who also have other concomitant itchy diseases, allergies, or scars constitution, or patients with induced urticaria will also be excluded.

#### 2.7.3 Termination and withdrawal criteria

Patients who fail to comply with the requirements of the research protocol will be terminated from study participation. This included patients who do not use the study drugs as prescribed. Participants with serious adverse events, disease exacerbation, or serious complications will be terminated from study participation. Those who cannot comply with the clinical protocol or are lost to follow-up or die during the study period will be also be removed from the study.

### 2.8 Study drugs

The experimental drug is danggui yinzi, and it will be provided by Sichuan Neo-Green TCM in a granular formulation prepared by Smart TCM Pharmacy (Sichuan, China). Danggui yinzi contains the following ingredients:

(1) Angelica (dang gui) 10 g
(2) Sheng (sheng di) 15 g
(3) Salvia miltiorrhiza (chuang xiong) 15 g
(4) Paeonia lactiflora (bai shao) 10 g
(5) Radix astragali (huang qi) 15 g
(6) Radix polygoni multiflori (fang feng) 10 g
(7) Radix polygoni multiflori (zhi shou wu) 9 g
(8) Tribulus terrestris (ji li) 10 g
(9) Bombyx silkworm (jiang chan) 10 g
(10) Glycyrrhiza (gan cao) 6 g

The herbs in the prescription are mixed and processed into a granular formulation and packaged into small, single-dose packs that will be distributed to the patients.

## 3 Interventions

### 3.1 Treatment plan

The 3 groups of patients, all diagnosed with blood deficiency and wind dryness syndrome according to the relevant TCM diagnosis and treatment guidelines, will take danggui yinzi mixed with about 150 mL of boiling water 3 times a day at 30 minutes after meals. Group A will also be administered with basic acupuncture as determined by a consensus of experts on the treatment of urticaria in TCM^[33]^: acupuncture Quchi (LI 11), Sanyinjiao (SP 6), and Xuehai (SP10). Group B will be administered shu-stream acupoints applied on the vigorous meridians of Qi and blood. According to accepted TCM theory, the shu-stream acupoints will be scheduled for specific times based on predictable, rhythmic disease exacerbations. For example, if CSU is active between 23:00 and 1:00, Zhlinqi (GB41), the shu-stream acupoints of the gallbladder meridian of foot-shaoyang will be applied.

### 3.2 Outcome measures

#### 3.2.1 Main efficacy index

Once a week, study participants will complete the measurement of 7-day urticaria activity score, which sums the daily hive count and itch severity recorded over 7 consecutive days.^[34]^ Quality of life will be evaluated using the chronic urticaria quality of life questionnaire.^[35]^

#### 3.2.2 Secondary efficacy index

Investigators will score the patients’ main symptoms using the TCM guidelines for clinical research of new drugs.^[36]^ The overall evaluation of the patients’ condition will be evaluated using the visual analogue scale.

### 3.3 Safety assessment

In China, Danggui yinzi has been used safely for more than 1000 years. The study dosage is the dosage recommended in the People's Republic of China Pharmacopoeia (2015 Edition). The acupuncture points selected for this study will all be located in the limbs. We will record any incidents of local hematomas, broken needles, residual needles, dizziness, unbearable acupuncture pain, severe pain after 1 hour, and local infection. In addition, to further evaluate the safety of this study, we will record the participants’ subjective observations and the results of laboratory tests, especially as they pertain to gastrointestinal intolerance and damage to the heart, liver, kidneys, and other organs from the time of enrollment through the follow-up period.

No matter whether the adverse events are related to the treatment methods of this study, they should be recorded in detail, including the occurrence time, symptoms, degree, duration, treatment methods and results, etc. In case of serious adverse events, the investigator must report to the clinical research responsible unit within 24 hours, and record it on case report form (CRF). At the same time, researchers will take necessary measures according to the condition and decide whether to stop the trial.

### 3.4 Statistical analysis

Study data will be analyzed by experts from the Statistics Teaching and Research Office of Chengdu University of TCM, using statistical analysis software package SPSS 22.0. All data will be expressed in terms of mean ± standard deviation. If the sample data are distributed normally and the variance is uniform, the Student *t*-test will be used. The paired sample *T*-test will be used for intra-group comparisons, and the independent sample *T*-test will be used for inter-group comparisons. If the variance is not uniform, the approximate *T*-test will be used to compare the mean of the 2 independent samples. If the sample data are not normally distributed, the nonparametric rank sum test will be used. Pearson Chi-squared test will be used to count the data. Statistical significance will be set at *P* < .05.

### 3.5 Data management

All records about patients will be collected in CRFs by trained and qualified investigators. Once a CRF is completed, the original record cannot be covered and changed if any modifications are made. The completed CRFs will be reviewed by the clinical inspector. The data will be input and proofread by 2 trained administrators independently to ensure the authenticity. After data review, the established database will be locked by researchers and statisticians until submitted to experts for statistical analysis. The Sichuan TCM evidence-based Medicine Center (Chengdu, China), which does not have any competing interests, will be responsible for monitoring the data. The Department of Science and Education of Chengdu Integrated TCM and Western Medicine Hospital, which is independent of the investigators, will perform data audits in the process of the trail.

## 4 Discussion

CSU is a common disease with a high recurrence rate. The average duration of CSU is about 2 to 5 years, and disease severity has been associated with disease duration.^[37]^ Although modern medicine can relieve the symptoms of CSU, current medications are with side effects, and there is currently no mean of preventing recurrence. Extended periods of disease can have a negative physical and psychological impact on patients and their families, and they can even affect the patient's ability to function socially. This, in turn, can affect the patients’ condition, which can form a vicious circle. Therefore, finding effective interventions that can treat CSU, reduce the frequency of recurrence, or prolong intervals of remission, continues to be a difficult problem in clinical practice.

Although TCM and acupuncture have often been used to treat CSU, clinician opinions differ on the best treatment, and there is no universally recognized therapy.^[38]^ Moreover, TCM treatment approaches often reflect theories propounded by different schools. Additionally, different methods are used to evaluate disease severity, and the level of evidence-based medicine recommendations is low. Therefore, we designed this RCT to evaluate the efficacy and safety of acupuncture combined with TCM herbal medicine in the treatment of CSU.

This study incorporates several innovations. The first is theoretical and is based on the TCM concept of time medicine, i.e. the rhythmic ebb and flow of Qi and blood in meridians and viscera in a 24-hour period. Based on the theory that “if the disease alleviated sometimes and aggravated sometimes, select Shu-Stream Points” in Huangdi Neijing, this study will use shu-stream acupoints to explore whether this method can improve the clinical symptoms of time-related CSU, to maximize the therapeutic effect. The study's proposed treatment approach is also innovative. The existing literature does not contain any report on the use of 5-shu acupoints to treat CSU. We expect that this method will become an accepted new therapy for CSU.

We recognize that this study design has some inherent limitations. It is being conducted in Sichuan, China, using an herbal drug formulation that is not widely available and a somewhat unfamiliar acupuncture approach, so we are not certain whether the results will be generalizable to other nationalities. Also, our follow-up time is relatively short. It is certain that no matter what our results are, further evaluation will be needed. Despite these limitations, we believe that this study will be beneficial to demonstrate the benefits of 5-shu point acupuncture in the treatment of CSU. In the future, we will include laboratory indicators for multidimensional evaluation and will carry out a wider range of multicenter RCTs. We expect that, because it is a simple, safe, efficient, inexpensive therapy, acupuncture treatment will become more popular and widely accepted.

## Author contributions

**Conceptualization:** Yuesi Qin, Jing Guo.

**Data curation:** Pan Song, Tianshu Hou, Wenxia Lin.

**Investigation:** Yan He, Ming Han, Qianying Yu.

**Methodology:** Yuesi Qin, Jing Guo.

**Project administration:** Hong Su, Mingling Chen.

**Resources:** Yuesi Qin, Jing Guo.

**Supervision:** Hong Su.

**Writing – original draft:** Yuesi Qin, Jing Guo.

**Writing – review & editing:** Mingling Chen.
